# Ninety‐day morbidity of robot‐assisted redo surgery for recurrent rectal prolapse, mesh erosion and pelvic pain: lessons learned from 9 years’ experience in a tertiary referral centre

**DOI:** 10.1111/codi.15979

**Published:** 2021-11-16

**Authors:** Emma M. van der Schans, Paul M. Verheijen, Ivo A. M. J. Broeders, Esther C. J. Consten

**Affiliations:** ^1^ Department of Surgery Meander Medical Centre Amersfoort The Netherlands; ^2^ Faculty of Electrical Engineering, Mathematics and Computer Science Institute of Technical Medicine University of Twente Enschede The Netherlands; ^3^ Department of Surgery University Medical Centre Groningen Groningen The Netherlands

**Keywords:** mesh erosion, pelvic pain, rectal prolapse, recurrence, ventral mesh rectopexy

## Abstract

**Aim:**

With increasing follow‐up of patients treated with minimally invasive ventral mesh rectopexy (VMR) more redo surgery can be expected for recurrent rectal prolapse, mesh erosion and pelvic pain. The aim of this study is to evaluate the 90‐day morbidity of robot‐assisted redo interventions.

**Method:**

All robot‐assisted redo interventions after primary transabdominal repair of rectal prolapse between 2011 and 2019 were retrospectively analysed and compared with the results for patients after primary robot‐assisted VMR during the same period. The redo interventions were divided into groups based on the indication for surgery (recurrent prolapse, mesh erosion, pelvic pain). Intraoperative complications and 90‐day postoperative morbidity were evaluated.

**Results:**

Three hundred and fifty nine patients were treated with primary VMR, with 73 for recurrent rectal prolapse, 12 for mesh erosion and 14 for pelvic pain. Complications of recurrent prolapse surgeries were comparable to those of primary VMR (*p* > 0.05). More intraoperative complications, minor and major complications were seen in redo surgery for erosion compared with primary VMR (23% vs. 3%, *p* = 0.01; 31% vs. 11%, *p* = 0.055; and 38% vs. 1%, *p* < 0.01 respectively). The frequency of intraoperative complications after redo surgery for pelvic pain was 7% with minor and major morbidity rates of 14% and 7% (*p* > 0.05). Half of the patients with pelvic pain experienced relief of their symptoms.

**Conclusion:**

Redo surgery for management of recurrent rectal prolapse is safe. Redo surgery for mesh erosion is associated with high morbidity rates. Redo surgery for pelvic pain can have major complications and is only effective in half of the cases.

**What does this paper add to the literature?**

This paper provides new information regarding the safety and risks of redo surgery for recurrence, mesh erosion and pelvic pain. It will help clinicians and patients in treatment decision‐making and preoperative counselling in both primary ventral mesh rectopexy and redo surgery after previous rectal prolapse surgery.

## INTRODUCTION

Since its introduction in the early 2000s minimally invasive laparoscopic ventral mesh rectopexy (VMR) has found wide acceptance in the treatment of rectal prolapse (RP) [1, 2]. Unfortunately, 5%–15% of patients develop recurrence during medium‐ to long‐term follow‐up [3, 4, 5]. Moreover, complications such as mesh erosion and infection (2%) or spondylodiscitis (0.2%) can occur [6, 7, 8]. This may require a redo intervention.

The majority of redo surgery is performed in patients who develop a recurrent RP. The treatment strategy for recurrent prolapse is not uniform and a previous attempt to create a treatment algorithm was not successful [9]. However, redo VMR seems feasible and is performed in several clinics. Another transabdominal approach, such as resection rectopexy, can be considered too, next to a perineal approach. Information on the outcome of redo surgery for recurrent RP is scarce and comes from small and heterogeneous cohorts [10, 11, 12, 13].

Patients with mesh erosion usually have symptoms of pain, dyspareunia, purulent or serosanguinous discharge and/or functional complaints [10, 14, 15]. Asymptomatic cases have also been described [15, 16]. In severe cases, mesh erosion can lead to a rectovaginal fistula or spondylodiscitis. Patients with mesh erosion form a second group that requires redo surgery to remove the mesh and close the defect. Treatment strategy varies between clinics and information on this type of surgery is scarce [10, 14, 15, 17, 18].

A third group consists of patients with therapy‐resistant pelvic pain, without evidence of mesh erosion, who request mesh removal. The incidence of pelvic pain is understudied and only recently a first retrospective study described de novo pelvic pain in 15% of patients [19]. Currently, there are no data on the outcome of redo surgery for therapy‐resistant pelvic pain [20].

In this study all robot‐assisted surgeries after previous surgery for RP in a tertiary referral centre were analysed. The aim was to evaluate the safety of redo interventions with regard to intraoperative complications and 90‐day morbidity in patients treated for recurrent RP, mesh erosion or pelvic pain.

## METHOD

### Study design

This study was conducted in a tertiary referral centre for pelvic floor pathology with a national referral function. The study protocol was approved by the institutional review board and requirement for informed consent was waived due to the retrospective study design. Part of the cohort has been described in previous studies with other research aims [4, 7].

All patients undergoing a robot‐assisted intervention related to RP between March 2011 and December 2019 were included. All abdominal procedures for primary and redo interventions were performed with the Da Vinci robotic system (Intuitive Surgical). A redo intervention was defined as surgery to treat a complication after a previous abdominal intervention for RP and/or apical prolapse. This includes redo surgery for (1) recurrent RP, (2) mesh erosion and (3) pelvic pain.

### Outcome parameters

Primary outcome measurements were intraoperative complication rates and 90‐day morbidity rates. Outcomes were compared with primary robot‐assisted VMR (RVMR). Major complications were defined as Clavien–Dindo (CD) classification grade >2 [21]. If patients suffered from more than one complication, the most severe complication was counted in the comparison with primary RVMR.

### Preoperative work‐up

Patients were assessed by a multidisciplinary team. Additional imaging and functional assessments were made on indication.

#### Recurrent rectal prolapse

The preoperative work‐up was similar to that of patients with primary complaints and has been described in detail previously [4]. Patients were counselled for redo RVMR or robot‐assisted resection rectopexy (RRR). An additional sacrocolpopexy was considered in cases of symptomatic multicompartment pelvic organ prolapse [16].

#### Mesh erosion

Physical examination of the rectum and/or vagina was performed in the case of complaints possibly related to mesh erosion. If the erosion was small and without signs of infection, transvaginal or transanal excision was considered. If this failed or did not seem appropriate (e.g. due to infection, size of the defect or fistula), a robot redo intervention was planned to remove the mesh and close the defect. The possible need for a diverting stoma was discussed.

#### Pelvic pain

In cases of chronic pelvic pain after previous VMR or other prolapse surgery with mesh implantation, other underlying causes of pain (e.g. mesh erosion/infection, anal fissure, haemorrhoids) were excluded. A redo intervention for mesh removal was performed with great reluctance and was considered if conservative pain management failed and patients requested mesh removal. Patients were counselled that surgery was not a guarantee of relief of complaints. Because of the risk of recurrence of prolapse symptoms after mesh removal, patients were additionally counselled for resection rectopexy.

### Surgery

#### Recurrent RP

Techniques and materials for redo RVMR were similar to primary RVMR [7]. Previously placed implants were removed when they prohibited optimal fixation of a new polypropylene mesh. In June 2019, a switch from nonabsorbable to absorbable sutures was made.

RRR was performed according to previously described techniques [22]. Old mesh implants were removed to prevent complications of mesh infection related to the potential risk of anastomotic leakage.

#### Mesh erosion

All mesh was intentionally removed, and the defect was closed with transabdominal (±omentoplasty) and transvaginal and/or transrectal absorbable sutures.

Depending on the location and extent of the defect, a diverting stoma was created in patients who were expected to have a high risk of development of a new rectovaginal fistula. In the case of pelvic pain all mesh and tackers were removed unless the risk of complications seemed higher than the possible gain.

All patients were routinely seen at 6–12 weeks after surgery in the outpatient clinic.

### Statistical analysis

Data are presented as mean ± standard deviation (SD) and median with interquartile range (IQR) for parametric and nonparametric continuous variables, respectively. Numbers and percentages are used for categorical variables. The independent samples *t*‐test, the Mann–Whitney *U*‐test and the chi‐square or Fisher's exact test were used as appropriate. A *p*‐value <0.05 was considered significant. Analyses were performed using SPSS version 24 (IBM Corp.).

## RESULTS

A total of 462 robot‐assisted surgeries were performed: 359 were primary RVMR cases and 103 were redo interventions. Of the redo interventions, the indication for surgery was a recurrent prolapse in 76 cases (72%), mesh erosion or infection in 13 cases (12%) and pelvic pain in 14 cases (13%). Baseline characteristics of patients with primary RVMR and patients with a redo intervention per subgroup are shown in Table 1. The history of prolapse surgeries is shown in Table 2. Fifty per cent of patients, equally spread amongst the indications for redo surgery, were external referrals.

**TABLE 1:** Baseline characteristics and complication rates of primary robot ventral mesh rectopexy cases and robot redo intervention cases

**Patient characteristics**	**Primary RVMR (N = 359)**	**Redo for recurrent RP (N = 76)**	***P*‐value**	**Redo for mesh erosion (N = 13)**	***P*‐value**	**Redo for pelvic pain (N = 14)**	***P*‐value**
Age (years), range	61 (50–69)	57 (46–70)	0.139	55 (51–68]	0.627	49 [44–58]	0.027
Female sex, *n* (%)	331 (92)	76 (100)	0.008	12 (92)	1.000	14 (100)	0.612
BMI (kg/m^2^), median [IQR]	24.6 [22.0–28.3]	25.4 [23.2–29.7]	0.022	22.6 [21.3–27.3]	0.380	25.6 [23.8‐29.2]	0.332
ASA grade, *n* (%)							
1	135 (38)	25 (33)	0.57	3 (23)	0.644	4 (29)	0.378
2	185 (51)	41 (54)		8 (62)		10 (72)	
3	36 (10)	10 (13)		2 (15)		‐	
4	3 (1)	‐		‐		‐	
**Complications and outcome of surgery**	**Primary RVMR (N = 359)**	**Redo for recurrent RP (N = 76)**	***P*‐value**	**Redo for mesh erosion (N = 13)**	***P*‐value**	**Redo for pelvic pain (N = 14)**	***P*‐value**
Intraoperative complications, *n* (%)	12 (3)	4 (5)	0.498	3 (23)	0.012	1 (7)	0.397
Postoperative complications (CD), *n* (%)							
Grades 1–2	40 (11)	6 (8)	0.403	4 (31)	0.055	2 (14)	0.664
Grades 3–4	5 (1)	3 (4)	0.149	5 (38)	<0.001	1 (7)	0.206
Mesh removal, *n* (%)							
Partial		4 (5)	‐	2	‐	4 (29)	‐
Complete		3 (4)	‐	11	‐	6 (43)	‐

*Abbreviations:* ASA, American Society of Anesthesiologists; BMI, body mass index; CD, Clavien–Dindo; IQR, interquartile range; *n*, number.

**TABLE 2:** History of pelvic organ prolapse surgeries of robot redo cases

**History of POP surgeries**	**Recurrent RP; N = 76**	**Mesh erosion; N = 13**	**Pelvic pain; N = 14**
RVMR/LVMR	55	12	13
RSCR/LSCR	8	1	1
RSC/LSC	8	1	
Open VMR	4	1	
Suture rectopexy	2		
Abdominal rectopexy with mesh (e.g. Ripstein)	6		2
Colporrhaphy with mesh	2	1	
Posterior rectopexy	1		
Subtotal colectomy with stapled rectopexy	1		
Delorme	3	1	
Median time in months between primary and redo surgery [ IQR]	40 [19–76]	29 [17–51]	23 [10–49]

*Abbreviations:* IQR, interquartile range; RVMR/LVMR, robot‐assisted/laparoscopic ventral mesh rectopexy; RSCR/LSCR, robot‐ assisted/laparoscopic sacrocolporectopexy; RSC/LSC, robot‐assisted/laparoscopic sacrocolpopexy.

### Recurrent rectal prolapse

**FIGURE 1:**
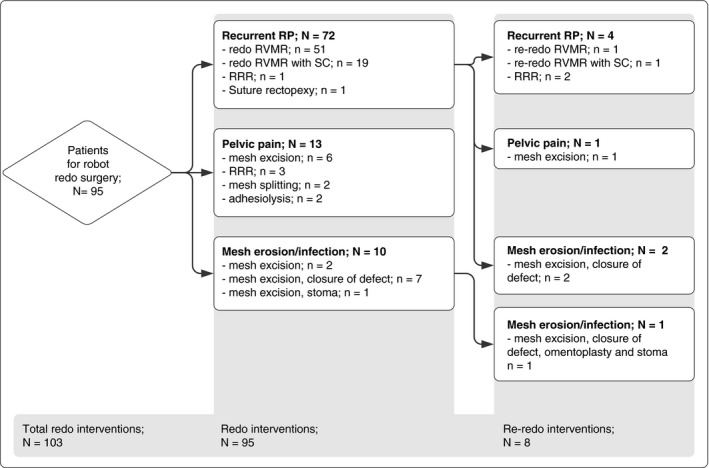
Flowchart of all robot redo interventions. Abbreviations: RP, rectal prolapse; RRR, robot resection rectopexy; RVMR, robot ventral mesh rectopexy; SC, sacrocolpopexy

Seventy six robot redo surgeries were performed for recurrent RP in 72 patients. Four patients underwent a second robot redo intervention due to a re‐recurrence (Figure 1). Redo RVMR was performed in 72 cases and was extended with a sacrocolpopexy in 20 patients. In one patient a suture rectopexy was performed because the rectovaginal septum was inaccessible, which prohibited the implementation of a new mesh. RRR was performed in three patients who has already been treated once before with a redo VMR and presented with a re‐recurrence.

A careful assessment of operation notes showed that failure of the previous VMR was thought to be related to suboptimal distal mesh positioning in 54 cases (71%), i.e. the mesh was not positioned deep enough in the rectovaginal septum, was positioned too laterally or failed to give adequate tension. In three cases (4%) the mesh was luxated from the sacral promontory. In seven cases (9%) reason for failure was unclear and 12 patients (16%) had a previous rectal suspension other than VMR (Table 2).

The intraoperative complication rate was 5%. Nine postoperative complications were observed, of which four were major complications (5%). Complications and their treatment are depicted in Table 3. There were no statistical differences in intraoperative complications and postoperative morbidity compared with primary RVMR (Table 1).

**TABLE 3:** Ninety‐day morbidity in robot redo interventions

**Indication for redo**	**Complication: management**	**CD classification**	**Frequency**
Recurrent prolapse	Wound infection: conservative wound management^a^	CD1	2
	Abnormal pain: painkillers	CD1	2
	Bleeding: suture and compression	CD1	1
	Asymptomatic recurrence: conservative management	CD1	1
	UTI: antibiotics^b^	CD2	1
	Atrial flutter: chemical cardioversion^b^	CD2	1
	Mechanical ileus: DLS without abnormalities^a^	CD3b	2
	Iatrogenic lesion previous DLS: laparotomy, closure lesion, omentoplasty, postoperative ICU admission^a^	CD4a	1
Mesh erosion	Wound infection: conservative wound management	CD1	1
	Electrolyte imbalance: supplementation	CD1	1
	Oedema of colostomy: canula^c^	CD1	1
	Paralitic ileus: NGT for decompression ± TPN^c/d^	CD2	2
	UTI: antibiotics	CD2	1
	Infection e.c.i.: antibiotics	CD2	1
	Decompensation: diuretics^c^	CD2	1
	Delirium: antipsychotics^e^	CD2	1
	Spondylodiscitis with fistula from rectum: prolonged antibiotics	CD3a	1
	Rectovaginal fistula: loop colostomy^d^	CD3b	2
	Rectovaginal fistula: residual mesh removal, omentoplasty, loopileostomy	CD3b	1
	Purulent peritonitis: lavage, omentoplasty, loop colostomy^e^	CD3b	1
Pelvic pain	Bleeding: suture and compression	CD1	1
	Urinary retention: catheter	CD1	1
	High‐output stoma: TPN and medical management^f^	CD2	1
	Rectal perforation: closure rectal wall defect and loop ileostomy^f^	CD3b	1
	Intra‐abdominal abscess: lavage	CD3b	1

Note

Six patients suffered from more than one postoperative complication. The combination of complications is indicated with superscript letters (a–f), where each letter represents an individual patient.

*Abbreviations:* CD, Clavien–Dindo; DLS, diagnostic laparoscopy; e.c.i., e causa ignota; ICU, intensive care unit; NGT, nasogastric tube; TPN, total parenteral nutrition; UTI, urinary tract infection.

After redo RVMR two patients developed a mesh erosion after the 90‐day postoperative course (after 22 and 82 months). These cases will be further elaborated on as part of the mesh erosion group.

### Mesh erosion

Twelve patients diagnosed with mesh erosion underwent a robot redo intervention (six vaginal erosions and six rectal erosions). In one patient a second robot redo intervention was necessary after 9 months because of a rectovaginal fistula due to infection of residual mesh (Figure 1). Only one patient was successfully treated with transrectal excision of an eroding suture. The median time between robot redo surgery and primary mesh implantation was 29 months (IQR 17–51 months; Table 2). Four patients had already had previous unsuccessful surgery to treat erosion (either transanal, transvaginal or laparoscopic) within the previous 6 to 26 months. In five patients (42%) the erosion seemed to result from nonabsorbable sutures eroding through the rectal or vaginal wall. Due to infection or fistula, two patients were given a loop ileostomy and one patient was given a diverting colostomy before the robot redo intervention.

An intraoperative complication occurred in three patients (23%), compared with 12 patients (3%) in primary RVMR (*p*‐value 0.01; Table 1). Four patients (31%) had one or more minor postoperative complications (versus 11% in primary RVMR, *p*‐value 0.06) and five patients (38%) suffered from a major complication (versus 1% in primary RVMR; *p*‐value <0.001). In four patients, major morbidity led to the creation of a loop ileostomy or colostomy (Table 3).

### Pelvic pain

Fourteen patients underwent a robot redo intervention due to pain resistant to medical management and assumed to be related to mesh insertion. Patients in this group were significantly younger than in the primary RVMR group (Table 1). In six patients the mesh was completely removed and in four patients it was partially removed. In seven patients the tackers were also removed. In three patients mesh excision was part of a RRR. In two patients the mesh gave too much tension on the rectal suspension, causing pain. In these cases, the mesh was split to release the tension. In two patients the mesh was released from the posterior vaginal wall because of strong adhesions. Two patients (14%) were diagnosed with a minor complication and one patient (7%) suffered from major complications which led to the creation of a loop ileostomy (Table 3). The difference in morbidity rates compared with primary RVMR did not reach statistical significance.

A review of medical records showed that in seven patients (50%) pain was relieved to the patient's satisfaction, six patients (43%) still experienced pain and in one patient it was unclear what effect surgery had had on the preoperative complaints. The pain relief experienced was better in the group with complete mesh removal (five out of six patients with improved symptoms) compared with patients in whom mesh removal was incomplete (two out of eight patients with improved symptoms).

## DISCUSSION AND CONCLUSIONS

Reasons for redo surgery after surgical repair for RP can be recurrence, mesh erosion or chronic pelvic pain resistant to conservative management. This study gives an overview of safety outcomes with regard to these three indications in patients undergoing robot‐assisted redo surgery.

Symptomatic recurrence after VMR has been described in 5%–15% of cases and increases with longer follow‐up [3, 4, 5]. This is the largest described cohort of patients treated for recurrent RP. Our results show comparable intraoperative and 90‐day postoperative complication rates compared with primary RVMR. This indicates that redo RVMR is a safe procedure in the treatment of recurrent RP. In three patients a RRR was chosen to treat recurrent RP. Although none of these patients experienced major morbidity, the small number of patients does not allow us to draw conclusions regarding the safety of RRR in treatment of recurrent RP. In our opinion this option should be considered nonetheless and, if opted for, performed by an experienced colorectal surgeon.

We found that in 71% of cases failure of the primary VMR was due to suboptimal fixation of the distal side of the mesh. This is important information for surgeons performing primary VMR and shows the importance of well‐trained and dedicated surgeons. Only a small proportion showed proximal mesh detachment. The change in our clinic to absorbable sutures was made only recently and does not affect this cohort. Although this change in material might conflict with our observation of suboptimal distal mesh fixation as a reason for failure of VMR, we reckon that by the time the suture material is absorbed, ingrowth of native tissue into the mesh implant will give sufficient support and fixation to prevent luxation. Future studies comparing recurrence of VMR with mesh fixed with absorbable sutures versus nonabsorbable sutures are important to confirm this hypothesis.

Laitakari et al. [13] recently described a retrospective cohort of redo VMR in 43 patients. Their major complication rate is comparable to the rate found in this study (5% vs. 5%). On the contrary, they reported a much higher frequency of proximal mesh detachment (30%). This could be due to differences in proximal fixation techniques (sutures versus tackers), but unfortunately their fixation method is not described. Three other studies have reported on the results of redo VMR for recurrent RP but are difficult to compare with our results due to other inclusion criteria and study endpoints [10, 11, 12].

Mesh erosion rates after VMR have been described in 1%–2% of patients [4, 6, 8]. In our opinion, all erosions should preferably be treated by complete mesh excision to prevent severe infection‐related complications such as fistula formation or spondylodiscitis. In selected cases it is possible to treat small mesh erosions with transanal excision, but these patients should be carefully monitored afterwards to make sure the erosion does not reoccur. This is one of the largest studies describing redo surgery for mesh erosion. This type of redo surgery proved to be associated with a high risk of intraoperative complications (23%) and postoperative minor and major morbidity (31% and 38% respectively). Compared with primary RVMR the differences were all (borderline) significant. Seven of the 12 patients needed a diverting ileostomy or colostomy, which are known to have a significant impact on quality of life. The observation of mesh erosion due to penetrating nonabsorbable suture material led us to change to absorbable material. This has been suggested by other clinics as well [6, 15].

There is no consensus regarding the treatment strategy for mesh erosion. This is reflected in the varying approaches described in previous studies. Management varies from transanal resection (and thus partial mesh removal) [6, 15, 18], to laparoscopic mesh removal [6, 10], to a combination of these two [14, 15], to resection of the affected organs [6, 10, 15].

Ratnatunga et al. [15] published their results on erosion management in 11 patients. They propose a step‐up approach where they start with examination under anaesthesia (±partial mesh excision). This can be followed by a laparoscopic procedure where the mesh is removed from the promontory but kept in place distally. After healing of the peritoneal defect, a third transanal procedure can be performed to remove the mesh. In their series only two patients (18%) had a diverting stoma. Although both our cohorts have small sample sizes, which makes comparison difficult, this lower frequency compared with our results (18% vs. 58%) is remarkable and could be due to various reasons. First, it could be they treated less severe erosions, since four patients were managed by examination under anaesthesia with partial mesh excision only. Secondly, they excluded two patients who were managed with low anterior resection during their early experience. On the other hand, it could be a result of their inventive step‐up approach which could be an example for others.

In the multicentre study of Evans et al. [6], 3 of 45 erosion patients were treated with anterior resection. In all patients with erosion in our cohort the rectum was spared. This could be a result of the high‐level of experience in our centre in combination with robot assistance. All in all, the high morbidity rates underline the requirement that this type of surgery should be performed by a dedicated team and referral to an experienced centre should be considered with a low threshold.

Although concerns about post‐VMR pelvic pain have increased, the prevalence of this complaint is understudied. Only recently a retrospective study was published describing de novo pelvic pain in 15% of patients after VMR [19]. Although the indication for redo surgery in cases of therapy‐resistant chronic pelvic pain is not straightforward, with the growing resistance to synthetic mesh we think it is important to publish on this type of surgery and its outcome. Most importantly, it should only be performed as a last resort and after careful patient counselling. This is the first study to evaluate surgery in patients with pelvic pain who opt for surgical excision of the mesh. The minor and major morbidity rates were 21% and 14%, respectively, versus 12% and 2% in primary RVMR cases, but these differences did not reach statistical significance. This could be due to the small number of patients being evaluated. Complete mesh removal was achieved in just 43% (6/14 patients) and pain relief in the 90‐day postoperative course was attained in just half of the patients. Patients with complete removal seemed to have better results with regard to postoperative pain relief. Previous studies have suggested that pelvic pain might be caused by scarring and foreign body reaction after the primary surgery and might persist after mesh removal [23]. Our findings of satisfactory pain relief in just half of the patients support this suggestion and underlie that redo surgery for pelvic pain should be reserved for selected cases and only be performed by an experienced surgeon after thorough patient counselling. Unfortunately, there was no standardized follow‐up to assess pre‐ and postoperative pain, and conclusions could only be drawn from notes in patient's records. Also, numbers of patients with successful conservative pain management are missing.

An overall limitation was the retrospective study design and the small cohorts of patients with redo surgery for mesh erosion and pelvic pain. However, this is still the largest group of patients to be described in the literature thus far. This study included patients up to 90 days postoperatively. Future studies should focus on functional outcome and quality of life after longer follow‐up.

In conclusion, redo surgery has been shown to be a safe procedure in the management of recurrent RP with comparable complication rates to primary VMR. In the management of mesh erosion, redo surgery is associated with a high major complication rate of 38%. Redo surgery for pelvic pain has a major complication rate of 7% and does not lead to relief of symptoms in a high percentage of patients. It should therefore be performed reluctantly. Based on these results, we strongly advise referral to a tertiary pelvic floor centre when redo surgery, and possibly even primary VMR, is being considered. In this way patients can be diagnosed and treated by an experienced and multidisciplinary team to minimize the risk of complications. This study is of great importance for clinicians and patients when considering both primary VMR and redo surgery for recurrence, mesh erosion or pelvic pain.

## CONFLICT OF INTERESTS

PMV, ECJC and IAMJB are proctors for Intuitive Surgical. EMvdS has no conflicts of interest to declare that are relevant to the content of this article.

## AUTHOR CONTRIBUTION

All authors qualify for authorship and agree to the submission.

## ETHICS APPROVAL

This retrospective study involving human participants was in accordance with the ethical standards of the institutional and national research committees and with the 1964 Helsinki Declaration and its later amendments or comparable ethical standards. The institutional review board of Meander Medical Centre approved this study.

## CONSENT TO PARTICIPATE

Requirement for informed consent to participate was waived by the institutional review board due to the retrospective nature of the study.

## Data Availability

The data that support the findings of this study are available from the corresponding author upon reasonable request.
